# Mononeuritis multiplex as a rare and severe neurological complication of immune checkpoint inhibitors: a case report

**DOI:** 10.1186/s13256-022-03290-1

**Published:** 2022-02-24

**Authors:** Safa Abdelhakim, Jonah D. Klapholz, Bhaskar Roy, Sarah A. Weiss, Declan McGuone, Zachary A. Corbin

**Affiliations:** 1 Department of Neurology, Yale School of Medicine, 15 York Street, New Haven, CT 06520 USA; 2 Department of Neurology, Yale School of Medicine, 333 Cedar Street, PO BOX 208028, New Haven, CT 06520-8028 USA; 3 Department of Medicine (Medical Oncology), Yale School of Medicine, 333 Cedar Street, New Haven, CT 06520 USA; 4 Department of Pathology, Yale School of Medicine, 310 Cedar Street, New Haven, CT 06520 USA

**Keywords:** Mononeuritis multiplex, Immunotherapy, Checkpoint inhibitors, Immune-related adverse events, Case report

## Abstract

**Background:**

Mononeuritis multiplex is a rare autoimmune peripheral neuropathy that typically presents in the context of vasculitis, diabetes, infection, or as a paraneoplastic syndrome. Adverse immune-related neurological conditions have been increasingly reported with the use of immune checkpoint inhibitors against cytotoxic T-lymphocyte antigen-4 and/or the programmed cell death protein 1/programmed death ligand-1 axis. Mononeuritis multiplex has only been reported twice from treatment of cancers with immunotherapy.

**Case presentation:**

Here we report a case of mononeuritis multiplex as a complication of immune checkpoint inhibitor therapy for melanoma. An 80-year-old non-Hispanic white female with recurrent melanoma was treated with combination ipilimumab and nivolumab and subsequently presented with progressive leg weakness, back pain, and difficulty ambulating. The diagnosis of mononeuritis multiplex was made, which was resistant to steroid pulses, chronic steroids, intravenous immunoglobulin, and rituximab. She developed progressive neurologic dysfunction and elected for hospice care. We found only two other cases reported in the literature.

**Conclusions:**

Increased awareness, prompt recognition, and aggressive treatments are likely the best opportunity for improved outcomes in this severe side effect.

## Background

Programmed cell death protein 1 (PD-1) and cytotoxic T-lymphocyte antigen-4 (CTLA-4) inhibitors are common immunotherapies that have been linked to adverse immune-related neurological conditions [1, 2, 4]. PD-1 and CTLA-4 play important roles in the fine tuning of T cell function to protect from potential threats while maintaining self-tolerance. Tumor cells take advantage of checkpoint negative regulation to evade immune surveillance. Anti-PD-1 (nivolumab, pembrolizumab) and anti-CTLA-4 (ipilimumab) agents have been found to unleash the antitumor immune response of the immune system. While rare, neurological toxicity during or following immunotherapy manifests in a wide variety of complications, including Guillain–Barré syndrome, chronic inflammatory demyelinating polyneuropathy (CIDP), myelitis, myasthenia gravis, and myositis. While immune-related adverse events (irAEs) typically respond to corticosteroids or other immunosuppressive agents, rare neurologic complications from checkpoint inhibitor therapy can rarely be refractory to multiple lines of immunosuppressive therapy [2–4].

The combination of nivolumab and ipilimumab is a standard first-line treatment for unresectable or metastatic melanoma [5, 6]. Despite the wide variety of neurological complications reported for cancer immunotherapies, mononeuritis multiplex (MM) in response to PD-1 inhibitor use for melanoma is a very rare complication, with only two reported cases in the literature [1, 7]. Here we report a case of steroid-refractory MM in which the response to intravenous immunoglobulin (IVIg) and rituximab was short lived.

## Case presentation

A 77-year-old non-Hispanic white female with history of a right carpal tunnel release and no family history of cancer was diagnosed with melanoma of the left ankle and treated with wide local excision. The melanoma recurred and she was started on ipilimumab and nivolumab. After three cycles she developed multiple irAEs including rash, transaminitis, and hypophysitis, all of which were responsive to steroids, as well as retinopathy requiring IVIg. Further anticancer therapy was held as she had a complete response to ipilimumab and nivolumab. She subsequently began to develop nonspecific symptoms of lower back pain. Several months later, she developed left lower extremity weakness and started to use a cane. Soon after, she also developed left foot drop. Magnetic resonance imaging (MRI) of the lumbar spine showed significant spinal stenosis at L4–L5, and she underwent L4–L5 posterior fusion without improvement in her symptoms. Her weakness continued to progress to include her left hand.

### Clinical findings

Per the initial neurologist’s evaluation, the patient’s mental status and cranial nerves were intact. The motor examination found atrophy in her left calf. Right arm showed intact strength, except for 4+/5 in the deltoid and thumb abduction. In the left arm, strength was intact except for 4/5 in finger extension, 4−/5 in finger abduction, and 4/5 thumb abduction. Right leg strength was intact, except for 4/5 in hip flexion. Left leg exam showed hip flexor strength of 4+/5, left foot dorsiflexion 2/5, left plantarflexion 4+/5, and left foot eversion and inversion 4/5. All modalities of sensory examination and finger–nose–finger testing were within normal limits bilaterally. Reflexes showed absent left ankle reflex, and otherwise 2+. Her gait was steppage, with evident left foot drop.

### Diagnostic assessment

Nerve conduction studies (NCS) and electromyography (EMG) showed severe reduction of response amplitudes of the left median and left ulnar motor studies, with relatively less involvement of the right median nerve. Motor responses of the left deep peroneal and tibial nerves were absent, and they were relatively spared on the right. Marked reductions of sensory response amplitudes were noted in the bilateral median and left ulnar nerves. Response amplitudes of the right radial nerve and bilateral sural nerves were normal. Overall, the EMG showed predominantly axonal involvement, and occasional relative slowing of conduction velocities in some of the nerves was felt to be secondary to cold extremity temperature and not indicative of a true demyelinating process. There was also a concern for bilateral carpal tunnel syndrome. Overall, the EMG and NCS demonstrated asymmetric involvement of multiple individual nerves in different extremities, without a length-dependent pattern (Table 1), consistent with MM.

**Table 1:** Motor and sensory nerve conduction study showing multifocal decreased compound muscle action potentials (CMAPs) as well as decreased sensory nerve action potentials (SNAPs)

		**DL (ms)**	CMAP (mV)	CV (m/s)	DL (ms)	CMAP (mV)	CV (m/s)
Motor nerve conduction studies		Left Median			Right Median		
Wrist	Abductor pollicis brevis	**6.4**	**0.5**		**4.6**	**3.3**	
Elbow	Abductor pollicis brevis		0.3	**32**		2.8	49
		Left Ulnar					
Wrist	Abductor digiti minimi	3.2	**1.4**				
Below Elbow	Abductor digiti minimi		1.4	57			
Above Elbow	Abductor digiti minimi		1.4	**38**			
		Left Peroneal			Right Peroneal		
Ankle	Extensor digitorum brevis	No response	No response	No response	4.2	3.3	
Fibular head					10.8	2.8	47
		Left Tibial			Right Tibial		
Ankle	Abductor hallucis	No response	No response	No response	5.3	**2.6**	
					12.7	2.1	45

			SNAP (µV)	CV (m/s)		SNAP (µV)	CV (m/s)
Sensory nerve conduction studies		Left Median			Right Median		
Wrist	Digit II		**11.0**	40		**12.5**	43
		Left Ulnar					
Wrist	Digit V		**4.4**	47			
		Left Radial					
Wrist	Anatomical snuff box		24.8	55			
		Left Sural			Right Sural		
Calf	Ankle		8.3	**35**		12.5	43

Bold represent values that are outside of the normal range for the nerve conduction laboratory

Laboratory findings included an elevated hemoglobin (Hgb) A1c, a positive antinuclear antibody (ANA) test of 1:160, and an antibody to fibroblast growth factor receptor-3 (FGFR-3) titer of 7000 (normal < 3000), which is known to be associated with inflammatory neuropathy. Serologic studies for anti-dsDNA (double-stranded DNA), anti-ribonucleoprotein (RNP), anti-Sjögren's Syndrome A (SSA), anti-Sjögren's Syndrome B (SSB), anti-Smith, anti-cyclic citrullinated peptide (CCP), anti-neutrophil cytoplasmic antibodies (ANCA), rheumatoid factor (RF), and a paraneoplastic panel were all negative. She also tested negative for *Treponema pallidum* antibody, Lyme antibody, Hepatitis B, Hepatitis C, and human immunodeficiency virus (HIV).

The patient’s symptoms, NCS, and EMG findings were consistent with MM, though we considered other diagnoses that could account for an asymmetric, sensorimotor, axonal neuropathy. Neurolymphomatosis (NL) is a dissemination of lymphoma to the peripheral nervous system, which can mimic inflammatory neuropathies such as MM. NL is diagnosed through cerebrospinal fluid (CSF) analysis, nerve biopsy, and MRI. In NL, CSF shows cells with lymphocytic predominance as well as protein elevation. Nerve biopsy is the gold standard for diagnosing NL. Fluorodeoxyglucose-positron emission tomography (FDG-PET) and MRI are also helpful, as they can show enlargement or enhancement of nerves or nerve roots [11]. Her nerve biopsy did not show any evidence of NL. Multifocal peripheral neuropathies may also result from metastatic invasion of the nerves, for which we found no evidence and would also be able to detect via nerve biopsy.

### Therapeutic intervention

The patient was started on IVIg 2 g/kg administered in two doses and methylprednisolone 1 g intravenous for five doses. She showed substantial improvement in her left-sided strength. She was started on a prednisone taper and received additional courses of IVIg. IVIg therapy was interrupted due to a hospitalization, and she noted worsening weakness. Given her lack of sustained response to prednisone and IVIg, she was started on rituximab.

### Follow-up and outcomes

**Fig. 1:**
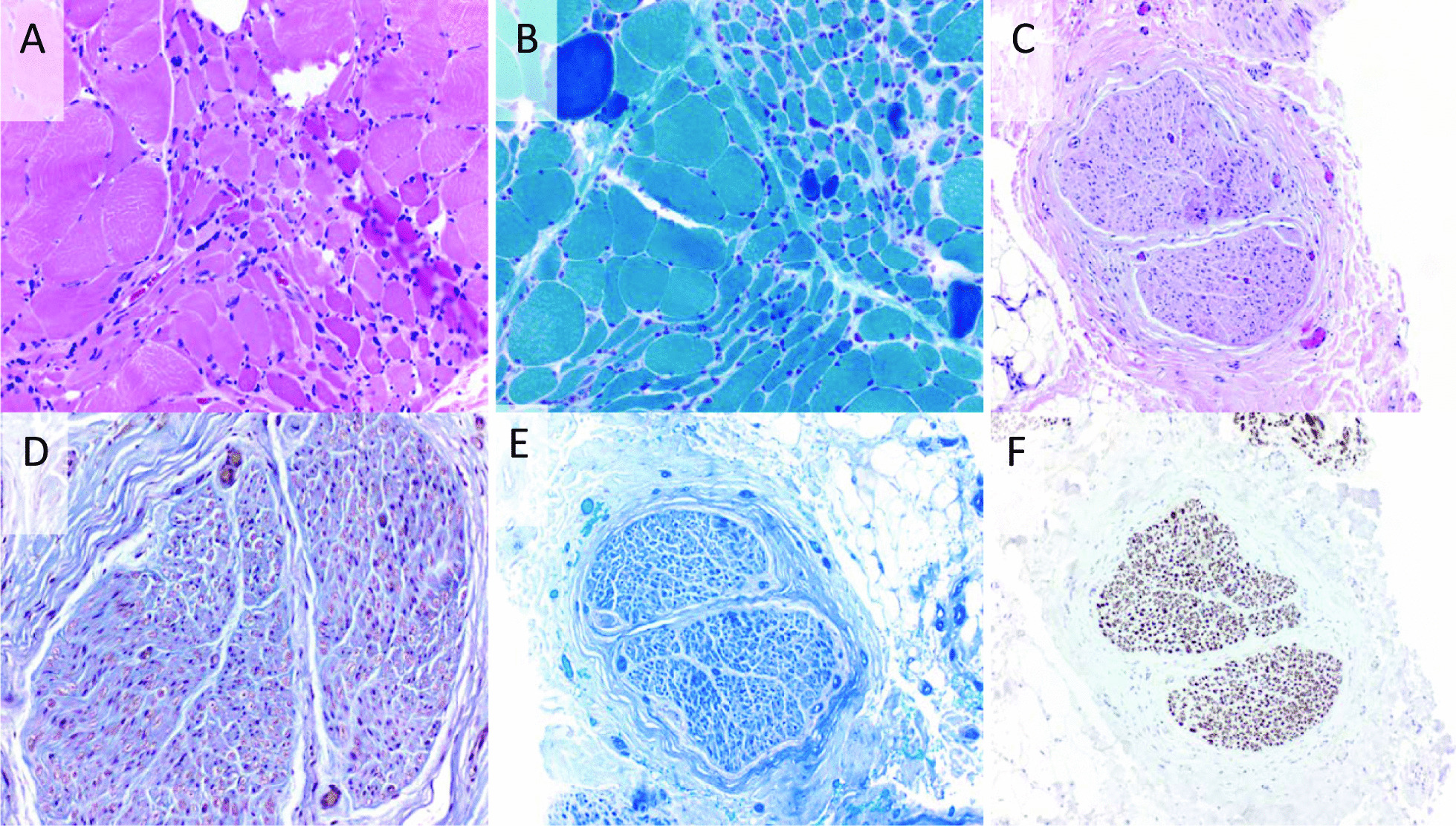
Muscle and nerve biopsy did not demonstrate evidence of vasculitis, granulomatous disease, or amyloid. Hematoxylin and eosin (H&E) stained section of skeletal muscle showing grouped atrophy (**A**); Gömöri trichrome highlights angulated atrophic muscle fibers (**B**); H&E stained section of peripheral nerve without vasculitis or significant inflammation (**C**); moderate endoneurial fibrosis on trichrome stain (**D**); intact myelin on Luxol fast blue stain (**E**); and preserved axons on neurofilament immunostain (**F**)

Following treatment with IVIg, repeat NCS/EMG confirmed progression of MM. Sural nerve biopsy showed endoneural fibrosis and scattered endoneural macrophages (Fig. 1). There was no evidence of fibrinoid necrosis or active vessel wall inflammation. There was no evidence of granulomas or amyloid on Congo red stain. There were rare scattered perivascular lymphocytes in the epineural connective tissue.

Despite sustained B-cell depletion, the patient developed severely progressive neuropathy with autonomic sequelae of urinary incontinence and constipation. The patient and her family elected to pursue hospice care, and she passed away.

## Discussion and conclusions

Upon a comprehensive literature review, we found two previously reported cases of MM in immune checkpoint inhibitor use [1, 4]. A detailed case report from Sakai *et al.* discusses a patient with metastatic melanoma who developed MM following immunotherapy treatment [1]. An 81-year-old man began experiencing limb weakness and sensory disturbance 8 days after beginning nivolumab monotherapy for mediastinal metastatic melanoma. He had proximal muscle weakness in all four limbs and left ulnar and bilateral peroneal palsies. Diagnosis was confirmed based on EMG findings [1]. He also had associated rhabdomyolysis. He was treated with solumedrol followed by oral prednisone, which halted the progression of motor weakness, and there was a recovery of grip strength. Dubey *et al.* made a brief mention of a case of ANCA-associated MM after immunotherapy, where a patient developed a sequential foot drop over several days followed by bilateral hand numbness and weakness and sensory ataxia. Electrodiagnostic studies showed evidence of mononeuritis multiplex [4].

PD-1 and CTLA-4 inhibitors have been associated with a variety of irAEs that most commonly affect the skin, gastrointestinal (GI) tract, liver, and endocrine system. Rates of grade 3 or 4 immune-related adverse events secondary to PD-1 inhibitor use are about 3–14% [8]. Neurological irAEs are less common but are being reported at an increasing rate (1.0–2.8%) [8]. These neurological events can occur at any point after the initiation of immunotherapy and involve a wide variety of neuropathies, neuromuscular disorders, and demyelinating polyradiculoneuropathies. To date, cranial neuropathies, non-length-dependent polyradiculoneuropathies, small-fiber/autonomic neuropathy, sensory neuronopathy, length-dependent sensorimotor axonal polyneuropathy, neuralgic amyotrophy, and mononeuritis multiplex have all been reported as adverse events in response to immune checkpoint inhibitors [4, 7].

MM is an asymmetric, asynchronous, sensory and motor mononeuropathy that involves damage in at least two separate peripheral nerve areas. It typically presents as the acute or subacute onset of multifocal sensory loss, weakness, and pain. It is usually associated with vasculitis, infections, diabetes, or paraneoplastic syndromes [9]. Diagnosing MM involves a combination of autoimmune serological assays and neurological testing. It is distinguished from demyelinating polyneuropathies through clinical presentation and EMG, indicating axonal asymmetric reduced sensory and motor action potentials [10].

The case demonstrated by Sakai *et al.* is similar to the one presented here in a few ways. Both patients had an EMG that showed reduced sensory nerve action potential (SNAP) and compound muscle action potential (CMAP) in a multifocal pattern. Moreover, recovery was not complete for either patient. The patient presented by Sakai *et al.* showed some response to treatment with corticosteroids but relapsed when dosage was decreased. Our patient was started on methylprednisolone and IVIg and showed worsening weakness in the setting of a prednisone taper and missed IVIg doses.

Checkpoint inhibitors are becoming increasingly common in treating cancers such as melanoma and, as a result, how they impact the nervous system requires continued investigation, with the goal of maximizing our scientific and clinical understanding of these treatments. Though there is no standard in treating neurological irAEs in immunotherapy, IVIg and plasmapheresis have been found to be effective in managing neurological sequelae of PD-1 inhibitors and CTLA-4 inhibitors [1, 2, 4]. In our case, the patient improved with IVIg and showed clinical and electrophysiological worsening in the setting of missed IVIg dosages. Immunosuppression was increased to rituximab with continued clinical progression and eventually an election to pursue hospice care.

Prompt suspicion, diagnosis, and therapeutic intervention provide the best opportunity for favorable outcomes for neurologic irAEs, however, the natural history of immune-mediated MM is variable. Considerations of re-challenging with immunotherapy are evaluated on a case-by-case basis, taking into account both the initial severity of irAEs and the clinical course.

## Data Availability

All data generated or analyzed during this study are included in this published article.

## Abbreviations

CIDP: Chronic inflammatory demyelinating polyneuropathy
CMAP: Compound muscle action potential
CTLA-4: Cytotoxic T-lymphocyte antigen-4
CV: Conduction velocity
DL: Distal latency
EMG: Electromyography
FGFR-3: Fibroblast growth factor receptor-3
irAEs: Immune-related adverse events
IVIg: Intravenous immunoglobulin
MM: Mononeuritis multiplex
NCS: Nerve conduction studies
NL: Neurolymphomatosis
PD-1: Programmed cell death protein 1
SNAP: Sensory nerve action potential
